# Do informal musical activities shape auditory skill development in preschool-age children?

**DOI:** 10.3389/fpsyg.2013.00572

**Published:** 2013-08-29

**Authors:** Vesa Putkinen, Katri Saarikivi, Mari Tervaniemi

**Affiliations:** ^1^Cognitive Brain Research Unit, Cognitive Science, Institute of Behavioural Sciences, University of HelsinkiHelsinki, Finland; ^2^Finnish Centre of Excellence in Interdisciplinary Music Research, University of JyväskyläJyväskylä, Finland

**Keywords:** music, brain development, event-related potential, training, auditory perception, informal musical activities

## Abstract

The influence of formal musical training on auditory cognition has been well established. For the majority of children, however, musical experience does not primarily consist of adult-guided training on a musical instrument. Instead, young children mostly engage in everyday musical activities such as singing and musical play. Here, we review recent electrophysiological and behavioral studies carried out in our laboratory and elsewhere which have begun to map how developing auditory skills are shaped by such informal musical activities both at home and in playschool-type settings. Although more research is still needed, the evidence emerging from these studies suggests that, in addition to formal musical training, informal musical activities can also influence the maturation of auditory discrimination and attention in preschool-aged children.

## INTRODUCTION

A multitude of experimental evidence shows that musical expertise has functional and structural manifestations in the brains of musically trained adult individuals. These can be observed in cortical and subcortical neural architecture underlying uni- and cross-modal sensory, motor, and cognitive functions (Münte et al., 2002; Jäncke, 2009). While the pioneering findings were correlational, indicating merely an association between the starting age of music training and enhanced brain functions (Pantev et al., 1998; Bengtsson et al., 2005), very recent longitudinal studies in children demonstrate a causal link between musical training and changes in brain structure and function (Putkinen et al., 2013b; Hyde et al., 2009; Moreno et al., 2009; Chobert et al., 2012).

In the great majority of these studies, musical training consisted of formal studies aiming at the mastery of one or more music instruments. However, for most young children, typical musical experiences consist of everyday musical activities such as singing, dancing, listening to recorded music, and musical play at home or in playschool-type settings. Furthermore, different computer and console games also attract children into “musical play” in an increasing manner (e.g., Singstar, Rockband, karaoke-based applications) and could be regarded as additional informal environments for music learning. Thus, there is an evident need for studies that examine informal musical activities as possible learning platforms in childhood.

## EARLY PERCEPTUAL PREREQUISITES FOR THE EFFECTS OF MUSICAL ACTIVITIES AND MUSICAL ENCULTURATION

Behavioral studies have demonstrated that already at around the age of six months infants are equipped with many of the perceptual and cognitive prerequisites for the putative beneficial effects of a musically enriched environment. Not only do they display fairly accurate discrimination of musically important basic sound features such as pitch (Olsho et al., 1987) and duration (Morrongiello and Trehub, 1987), but they are also sensitive to some more abstract aspects of musical sounds. For example, infants appear to encode melodies and rhythms in terms of relative pitch (Trehub et al., 1984) and relative duration (Trehub and Thorpe, 1989), are able to group individual tones by pitch (Thorpe et al., 1988), and show long-term memory for musical pieces (Plantinga and Trainor, 2005). More recently, event-related potential (ERP) studies have shown that musically relevant auditory abilities such as the neural discrimination of different intervals (Stefanics et al., 2009), sound grouping (Stefanics et al., 2007), perception of the missing fundamental (He and Trainor, 2009), auditory stream segregation (Winkler et al., 2003), and detecting the beat of rhythmic sounds (Winkler et al., 2009) are present already before the age of six months or even at birth. These early perceptual skills are also put to heavy use during childhood: young children typically receive ample musical exposure (Trehub, 2006) and appear to find music both interesting and enjoyable (Nakata and Trehub, 2004; Zentner and Eerola, 2010). Therefore, everyday musical activities are a rich source of experiences that may have the potential to shape auditory skill development.

Indeed, in behavioral studies even musically untrained adults show (implicit) competence in processing some fairly nuanced aspects of music in a way that is consistent with learning through mere incidental exposure (Cuddy and Badertscher, 1987; David Smith et al., 1994; Bigand and Pineau, 1997; Honing and Ladinig, 2009). ERP studies indicate that the brains of non-musicians automatically process some aspects of Western tonality and harmony (Koelsch et al., 2000, 2003; Brattico et al., 2006; Krohn et al., 2007). Presumably, these idiosyncrasies of Western tonal music are internalized by non-musicians through everyday musical experiences. Native language learning offers a well-known parallel example of such an exposure effect where, during the first year of life, the auditory system starts to tune to the speech sounds of one’s native language while simultaneously losing the sensitivity to non-native speech sound contrasts (Kuhl et al., 1992; Cheour et al., 1998). In infants, a development reminiscent of the tuning to native speech sounds appears to take place with regard to the processing of culturally typical vs. atypical metric (Hannon and Trehub, 2005) and scale structures (Trehub et al., 1999) in music. Consequently, adults show an advantage in processing music that follows the conventions of their culture (Kessler et al., 1984; Krumhansl et al., 2000; Drake and Ben El Heni, 2003; Demorest and Osterhout, 2012). At the very least, this enculturation process demonstrates that ambient exposure to music without specific training is sufficient for learning culture-specific implicit musical knowledge. More generally, these effects raise the question of whether informal exposure to music might also influence the development of auditory processing outside of the musical domain.

## MUSICAL ENRICHMENT IN EARLY CHILDHOOD

In light of the evidence reviewed above, it seems plausible that everyday musical activities during childhood such as exposure to parental singing and musical play by the child might influence auditory skill development. Yet so far only a few research endeavors have directly examined how variation in the amount of such activities is reflected in early auditory abilities. These will be introduced next.

**FIGURE 1:**
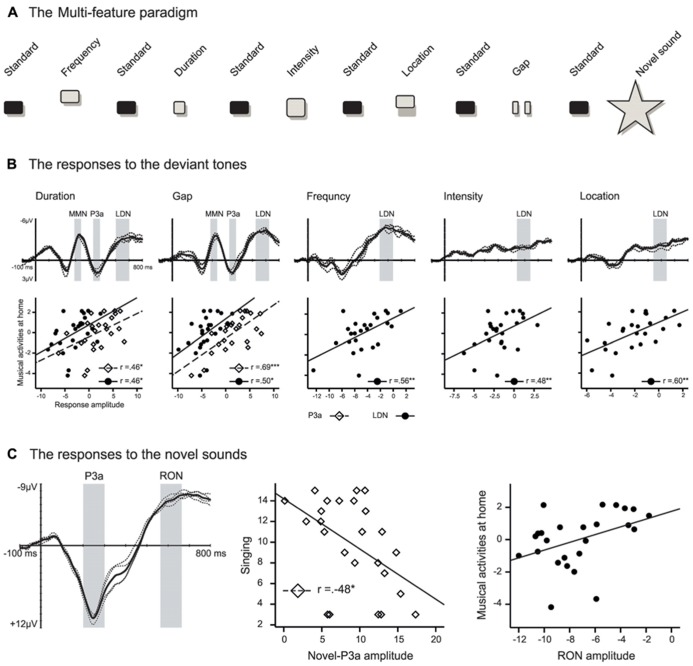
**(A)** Illustration of the multi-feature paradigm used in Putkinen et al. (2013a). In this paradigm standard tones (*P* ~ 0.50) alternate with deviant tones (*P* ~ 0.42) from five categories and novel sounds (*P* ~ 0.08). See Putkinen et al. (2013a) for details. **(B)** and **(C)** illustrate difference signals for the deviant sounds and novel sounds, respectively, and the scatter plots illustrating the correlations between the P3a (diamonds) and LDN/RON (dots) amplitudes and informal everyday musical activities. In the ERP figures, the thin dashed lines represent the difference signal at individual fronto-central channels and the thick solid line is the average of these signals. The gray bars indicate the latency windows that were used to calculate the response mean amplitudes. Panels **(B)** and **(C)** reproduced with permission from Putkinen et al. (2013a).

Recently, Putkinen et al. (2013a) set out to investigate whether the amount of informal musical activities is related to electrophysiological correlates of auditory change detection and attention in 2–3-year-old children. They used the *multi-feature paradigm* (Näätänen et al., 2004) to record several auditory ERP responses in parallel: the mismatch negativity (MMN), P3a, late discriminative negativity (LDN), and reorienting negativity (RON). These brain potentials reflect successive stages of auditory processing particularly in childhood. The MMN is thought to reflect memory-based discrimination of sound changes (Näätänen et al., 2007) while the P3a is an index of an ensuing involuntary attention shift toward such changes (Escera et al., 1998). The LDN, which is often seen in children but less so in adults, is related to further processing of sound changes but its exact functional role remains unclear (Bishop et al., 2011). Finally, the RON reflects attentional reorienting after distracting sounds (Schröger and Wolff, 1998). These ERP responses were recorded within a single sound sequence to changes in frequency, duration, intensity, perceived sound-source location, and the temporal structure of the sounds (i.e., infrequent silent gaps in sounds) and to surprising novel sounds (e.g., animal sounds; see **Figure 1A**).

In addition to ERP recordings, parents were enquired about how often their children engaged in different types of musical activities at home (e.g., singing and dancing) and how often the parents interacted with them musically (e.g., how often they sang to their children). A composite score indexing the amount of such musical everyday activities was found to be significantly correlated with the ERP response amplitudes (see **Figures 1B, C**). Firstly, a high amount of musical activities was associated with enlarged P3a responses to duration and gap deviants. This result suggests that the attention of the children from the more musically active families was more readily drawn toward the changes in duration and temporal structure of sound. Therefore, musical activities at home might affect how children consciously discriminate changes in temporal aspects of sounds. Second, high scores for the musical activities index were also associated with a diminished LDN across all five deviant types. The LDN is typical for immature auditory change detection since it decreases in amplitude with age as the brain matures (Hommet et al., 2009; Bishop et al., 2011). Therefore the reduced amplitude of the LDN in children with more musical activities at home suggests more mature auditory processing in these children.

The P3a elicited by the novel sounds, in turn, correlated with paternal singing so that the more the fathers reported singing to their children, the smaller the P3a elicited by the novel sounds was. Finally, the RON responses to the novel sounds were smaller in amplitude in children with high scores in the musical activity index. The P3a and RON responses elicited by novel sounds are regarded as indices of distractibility in children. The large amplitude of these responses is associated with behavioral distraction by the eliciting sounds (i.e., prolonged reaction times and/or decreased hit rates in a concurrent task that requires responding to stimuli unrelated to the distracting sounds) and the P3a is enlarged in children with attention deficit hyperactivity disorder (ADHD; Gumenyuk et al., 2005). Therefore, the reduced P3a and RON responses found by Putkinen et al. (2013a) in the children with more musical activities at home suggest that these children were less easily distracted by the novel sounds than children from less musically active home environments.

These results are interesting since they suggest that in early childhood, informal musical activities might affect the development of auditory skills essential for normal language development as well as attention skills that may be related to later school performance. Obviously, the correlational data of Putkinen et al. (2013a) do not allow one to conclude that the relation between everyday musical activities and response amplitudes was causal. However, several lines of evidence suggest that such an interpretation of the results is at least plausible. Firstly, animal studies indicate that early enrichment of the sound environment affects the organization of auditory cortices (Zhang et al., 2001; Engineer et al., 2004). Secondly, emerging evidence from experimental studies reviewed below support the notion that the kind of musically enriched early experience examined by Putkinen et al. (2013a) can influence the development of auditory processing.

In an ERP study, Trainor et al. (2011) randomly assigned 4-month-old infants either to a group that was exposed to recordings of melodies in a guitar timbre or to a group that heard the same melodies in a marimba timbre. After a week-long 20-min-per-day exposure to one of the two timbres, the guitar-exposed infants showed a larger obligatory response to guitar tones than to marimba tones whereas the opposite response pattern was found for the marimba-exposed group. Furthermore, occasional pitch changes in the guitar tones elicited a mismatch response only in the guitar-exposed infants whereas pitch changes in the marimba tones did not elicit a significant mismatch response in either group. These results suggest that in infants a relatively short exposure can strengthen the neural representations of a given timbre which is further reflected in enhanced processing of pitch in that timbre.

Gerry et al. (2012) randomly assigned 6-month-old infants either to infant-directed music classes based on the Suzuki method or to classes during which infants interacted with their parents while recorded music was played in the background, or to a control group with no lessons. After a 6-month follow-up, the infants took part in an experiment during which a piece of classical music and an atonal version of the same piece was played to them. Looking-time measurement indicated that the infants who had attended the Suzuki music classes preferred the original piece over the atonal version whereas the other two groups showed no preference. The authors interpreted this result as an indication of earlier learning of Western tonality in the Suzuki music group than in the control groups. Along the same lines, another study by the same team found that guided musical group activities with emphasis on infants and parents moving to music were associated with heightened sensitivity to the typical metric structure of Western music in 7-month-old infants (Gerry et al., 2010).

Although there are some clear differences between the aforementioned studies in the type of musical exposure, they all involve activities that parents spontaneously engage in with their children in order to musically enrich their auditory environment (i.e., moving to music, listening recorded music, singing etc.). One especially important common feature is that – in clear contrast to traditional studies looking at the neuroplastic effects of musical training – these studies examined musical experience that involved no formal training on a musical instrument (although in Gerry et al., 2012 the Suzuki classes reportedly involved playing percussive instruments). Also, in the studies of Putkinen et al. (2013a) and Gerry et al. (2012), social interaction between the parents and the child appeared to be a central mediator of the link between the musical experience and children’s auditory skills: In both studies the effects were specific to active and interactive musical behaviors whereas no effects were observed for more passive exposure to music. Together these studies suggest that even without formal instrument training, musical exposure may shape auditory development not only in the musical domain but also more generally by influencing basic auditory discrimination abilities and auditory attention.

Importantly, the positive effects of informal music activities are not limited to children with typical development. Recently, Torppa et al. (2012) investigated children (aged from 4 to 13 years) who were born deaf but who had received a cochlear implant in childhood. MMN and P3a responses particularly to timbre and pitch changes in musical multi-feature paradigm consisting of instrumental sounds were modulated by the singing activities of these children (Torppa et al., unpublished data). Cochlear-implanted children who sang regularly had earlier MMNs and P3as to frequency changes as well as earlier MMNs for changes from piano to cembalo timbre than cochlear-implanted children who did not sing. This finding is promising since it gives strong evidence against traditional views that cochlear-implanted individuals are not able to perceive and appreciate music. Instead, they can perceive and even produce music. Whether their readiness and willingness to sing facilitate neural sound change discrimination or whether it is the outcome of originally facilitated neural discrimination remains to be clarified in future studies.

## CONCLUSIONS AND FUTURE DIRECTIONS

In conclusion, the studies reviewed above suggest that musically rich environments might have beneficial effects on auditory abilities in childhood. We suggest that these effects are not specific to the musical domain but that informal musical activities might promote more general enhancement of auditory processing. Enhancement of these skills may have important consequences for example on the later development of language and attention.

Social aspects of musical activities probably play a key role in some of the effects of musical experience on the development of auditory skills. Not only do most of the musical activities that young children engage in – for obvious reasons – take place in social situations, but social interaction *per se* is probably a profoundly important component of early musical experience. Kirschner and Tomasello (2010) directly compared drum tapping to a rhythm in social and non-social situations in preschool-aged children and found more accurate spontaneous synchronization when drumming together with an adult than with a machine or a prerecorded beat. Moreover, the finding that social interaction (vs. passive exposure) appears crucial for native speech sound learning (Kuhl et al., 2003) implies that social interaction could facilitate early perceptual learning in the musical domain as well.

Overy and Molnar-Szakacs describe music as a primarily social experience that involves understanding the intentions behind motor actions that are required to produce musical signals (Molnar-Szakacs and Overy, 2006; Overy and Molnar-Szakacs, 2009). They suggest that this process relies on the mirror neuron system (MNS) and that music making could promote positive social interaction precisely because of the engagement of this system. Mirror neurons are generally thought to encode motor goals and intentions and thereby support action understanding, social learning, and interaction (Van Overwalle and Baetens, 2009; Bonini and Ferrari, 2011; however see, Cook et al., in press; Hickok, 2013). Whether the MNS is involved or not, positive social behavior has indeed been connected to music-making. For example, Kirschner and Tomasello (2010) found that children more often showed prosocial behavior after joint music making than after non-musical cooperation. In contrast to the view that supposes inborn mirroring properties with evolutionary origin (Bonini and Ferrari, 2011), the associative learning account holds that the capability of mirror neurons to match observed and executed actions is only acquired through experience (Heyes, 2010; Catmur, 2013). Thus, interactive music-making between the parent and the child can either be thought to be supported by the MNS or as a naturally engaging learning platform for developing the matching properties of these neurons.

There is a growing interest toward incorporating informal musical activities in clinical interventions for various conditions. Partly fuelling this interest, a pioneering randomized clinical study showed that everyday music listening can support cognitive recovery after stroke (Särkämö et al., 2008). In this study, stroke patients who were assigned to a group that listened to self-selected music for at least 1 h a day for 2 months showed greater recovery in verbal memory and focused attention than patients in an audio-book listening group or a control group and had lower depression and confusion scores relative to the control group. Clearly, these are highly encouraging findings with regard to stroke rehabilitation that again indicate that everyday musical activities can have deep and wide ranging effects on the brain.

Perhaps informal musical activities in childhood could also be harnessed to tune basic auditory processing. This seems especially relevant for disorders of language development, since several studies indicate that auditory discrimination in infancy predicts later language skills (Molfese, 2000; Molfese et al., 2001; Benasich and Tallal, 2002; Guttorm et al., 2005) and that basic auditory dysfunction might be a key feature of dyslexia (e.g., Tallal and Gaab, 2006). Furthermore, the attention-related effects found by Putkinen et al. (2013a) have similar implications for the normal and disturbed development of attentional control while the results of Torppa et al. (2012) suggest that the recovery of hearing in children with cochlear implants might be supported with musical activities.

In contrast to some non-musical everyday activities that have been suggested to have beneficial effects on brain development (e.g., sports), music may have an especially strong motivational component already very early in life and might suit the perceptual and motor capabilities of young children particularly well. Musical activities might therefore be in a special position to shape the brain during the early period of heightened neuroplasticity. Future studies should investigate whether this is true only for early childhood, i.e., whether there is an early sensitive period for these effects and whether these periods are relatively fixed or malleable by experience (for example, Kuhl, 2004, 2011 has suggested that exposure to more than one language in infancy could extend the sensitive period for native speech sound learning). Thus, longitudinal or large-scale cross-sectional studies starting from infancy need to be carried out in order to map the stability of the association between informal musical activities and auditory skills in childhood as well as their implications for later auditory development. Importantly, experimental intervention studies are needed to disentangle the direction of causality in these associations and to test the feasibility of incorporating everyday musical activities in treating and preventing impairments of auditory processing.

Taken together, the current findings suggest that music as a part of daily informal activities may improve several neurocognitive functions and thereby encourage the use of music in various educational settings such as daycare and school for pupils with and without special needs (see, e.g., Uibel, 2012). By reviewing the benefits that music even without formal instrumental training may offer for the modulation of basic neurocognitive functions in children, the current paper hopefully opens new avenues for future studies on the effects of informal musical activities on brain development.

## Conflict of Interest Statement

The authors declare that the research was conducted in the absence of any commercial or financial relationships that could be construed as a potential conflict of interest.
